# Impairment of human spatial orientation in the horizontal, but not the vertical plane, due to aging, cognitive decline, or chronic peripheral vestibular loss

**DOI:** 10.3389/fnagi.2025.1671562

**Published:** 2025-10-09

**Authors:** Johannes Gerb, Vivien Oertle, Burak Onmus, Marianne Dieterich, Thomas Brandt

**Affiliations:** ^1^German Center for Vertigo and Balance Disorders, LMU University Hospital, LMU Munich, Munich, Germany; ^2^Department of Neurology, LMU University Hospital, LMU Munich, Munich, Germany; ^3^Graduate School of Systemic Neuroscience, LMU Munich, Munich, Germany; ^4^Munich Cluster for Systems Neurology (SyNergy), Munich, Germany

**Keywords:** ageing, spatial orientation, vestibulopathy, pointing task, dementia, allocentric, egocentric, spatial cognition

## Abstract

**Introduction:**

Humans and other vertebrates exhibit anisotropic orientation and navigation skills, with better performances in the horizontal compared to the vertical plane. While horizontal navigation accuracy decreases with higher age, less is known about age effects on vertical spatial abilities. The same is true for disorders which cause spatial memory deficits, e.g., cognitive impairment or chronic peripheral vestibular loss.

**Methods:**

In this study, patients presenting at a tertiary centre for vertigo and balance disorders underwent a bedside test for spatial orientation abilities (3D real-world pointing task, 3D-RWPT), neurological and neuro-otological testing, and a cognitive screening (MoCA). The 3D-RWPT includes a spatial memory paradigm before and after a passive whole-body yaw-axis-rotation, which requires a (cognitively demanding) mental transformation of one’s body position relative to the targets, and the correct integration of vestibular sensory information. To assess the influence of each variable on pointing accuracy, a mixed linear regression model was used.

**Results:**

In total, 569 patients (302 females; mean age 62.77 ± 14.13 years) were included. In all paradigms of the 3D-RWPT, age constituted a highly significant predictor for angular inaccuracy in the horizontal plane (*p* < 0.001***), but not in the vertical plane (n.s.). MoCA-scores (patients with cognitive impairment: *n* = 178) showed significant impact on the horizontal accuracy in the transformation paradigm (*p* < 0.001***). In the postrotation task, bilateral peripheral vestibular dysfunction (*n* = 67) was a highly significant predictor for impaired horizontal (*p* < 0.001***) but not vertical (n.s.) accuracy. Male patients generally outperformed female patients in both planes.

**Discussion:**

Aging, cognitive decline, or chronic peripheral vestibular loss selectively impair human spatial orientation in the horizontal, but not the vertical plane. A possible explanation is that vertical and horizontal orientation in ground-based species use separate operational modes of spatial memory encoding. The continuous updating of the internal model of the body in space is realized through egocentric representation in the vertical plane and allocentric, that is world-based, representation in the horizontal plane. Additionally, heterogenous ageing patterns of relevant brain structures, and training effects could play a role.

## Introduction

Experimental evidence suggests that ground-based species, like vertebrates [e.g., rats (Jovalekic et al., 2011), dogs (Brandt and Dieterich, 2013) and humans (Zwergal et al., 2016)], perform better in situations requiring horizontal navigation compared to those testing vertical navigation. Based on these observations, previous researchers have identified a kind of physiological anisotropy in human spatial abilities—essentially a disparity in spatial orientation between the horizontal and the vertical planes (Zwergal et al., 2016). At a cellular level in rodents, anisotropic encoding has been observed, possibly contributing to this performance difference (Haymann et al., 2011). The activation patterns of place cells in the hippocampus and grid cells in the entorhinal cortex, as well as in the pre- and parasubiculum, diverge between horizontal and vertical navigation. Position-dependent activity was mainly elicited during horizontal navigation but largely absent when it came to vertical navigation (Haymann et al., 2011). Aiming to integrate the findings from the cellular to the systems level, different spatial representation modes have been postulated across the hippocampus and entorhinal cortex as the basis for three-dimensional spatial encoding, which involves re-mapping a quasi-planar spatial representation depending on the plane of locomotion (Jeffery et al., 2013). Although the neuronal networks for both planes partially overlap in the hippocampus, unique areas such as the retrosplenial or the visual cortex are more relevant for horizontal navigation, while vertical navigation is associated with stronger activations in the flocculus and the vestibular multisensory cortex (Zwergal et al., 2016). Unlike horizontal navigation, vertical navigation is less reliant on visual cues and landmarks in humans (Zwergal et al., 2016).

Thus, three-dimensional spatial navigation includes various brain areas, and the accuracy in experimental navigation paradigms typically declines throughout lifetime. These age effects are well known for horizontal navigation in humans (Moffat, 2009), non-human primates (Rapp et al., 1997), dogs (Head et al., 1995), and mice (Bach et al., 1999), but there are noticeable interindividual differences; potentially, this could be a sign of heterogeneous brain ageing patterns, where substantial variability has been demonstrated in large-scale neuroimaging studies (Yang et al., 2024).

With the lack of studies specifically investigating age-related decline of vertical spatial abilities, it is currently unknown how higher age affects vertical and horizontal spatial abilities differently. Furthermore, studies investigating the role of the vestibular system for spatial orientation as well as human navigation trials in dementia patients predominantly focus on the horizontal plane (Plácido et al., 2022). In this study, we therefore collected and analysed experimental data from a three-dimensional bedside test of spatial abilities [three-dimensional real-world pointing task, 3D-RWPT (Gerb et al., 2023a)] conducted in a clinical patient cohort. We aimed to investigate three main research questions: first, we wanted to assess if there is evidence of an age effect on vertical spatial abilities; second, we aimed to determine the differential influence of cognitive decline on horizontal versus vertical spatial accuracy in the 3D-RWPT; lastly, we wanted to analyse how chronic peripheral vestibular deficits (unilateral, UVP, or bilateral peripheral vestibulopathy, BVP) influence horizontal versus vertical orientation performance. For this, a total of 569 patients were enrolled.

The three-dimensional real-world pointing task (3D-RWPT) (Gerb et al., 2023a) was chosen as a simple and fast measure of spatial orientation abilities. In this test, patients are seated in front of well-visible targets and perform whole-arm pointing movements toward these targets in different body positions. This approach has proven to be reliable in healthy subjects (Gerb et al., 2022) and allows the inclusion of patients with cognitive impairment, since pointing is a universal and well-preserved method of interacting with peripersonal space. Even patients with severe dementia understand the test instructions, which is often a limiting factor in more complex experimental setups like virtual reality (VR) (Siette et al., 2024; Roberts et al., 2019). Furthermore, the 3D-RWPT setup integrates naturalistic aspects of daily locomotion in the horizontal plane, using input from both the otoliths and semicircular canals, rather than providing an artificial laboratory setting with unphysiological stimuli (which can be the case in VR experiments). This test therefore seemed especially suitable to investigate the role of lacking peripheral vestibular input for spatial navigation, which is still only partially understood (Yoder and Taube, 2014; Zwergal et al., 2024; Brandt et al., 2005; Kremmyda et al., 2016; Dobbels et al., 2019). However, it has been repeatedly shown that patients with chronic bilateral peripheral vestibulopathy suffer from an impairment of spatial memory and navigation. Previous research using the same testing setup had revealed distinct spatial impairment patterns in patients with chronic bilateral vestibular loss, who especially struggled in one subtest of the 3D-RWPT requiring the correct integration of vestibular-sensory information after a whole-body yaw-axis rotation without visual feedback, while patients with cognitive impairment showed lower accuracy in the subtest which encompasses mental transformation due to a changed body position relative to the targets (Gerb et al., 2024a). Importantly, the 3D-RWPT measures vertical and horizontal angular deviations of the pointing towards the respective targets separately. This approach appeared well-suited to access age effects and disease-specific reductions in spatial orientation. If the limitations in spatial orientation for the three experimental conditions of the 3D-RWPT (spatial memory, mental rotation, real body rotation) primarily affect the horizontal plane, this would have implications for clinical testing of both healthy individuals and patients, with further insight into the plane-specific internal representation of the body in space.

## Materials and methods

### Patients

729 patients presenting at a tertiary center for vertigo and balance disorders between 10/2020 and 10/2024 were screened for inclusion eligibility. Patients underwent a neurological examination, neuro-otological testing, a neuro-orthoptic assessment, a depression screening test (PHQ-9) (Kroenke et al., 2001), a cognitive screening using the Montreal Cognitive Assessment (MoCA) (Nasreddine et al., 2005) with the standard cutoff value of 26 points after correction for patient education, and the 3D-RWPT. For sample size calculation, G*Power (V3.1) was used (Faul et al., 2007). For a linear regression model based on a medium effect size of f^2^= 0.15, five tested predictors (MoCA, PHQ-9, age, sex, and vestibular function) and a power of 0.95, the critical F was calculated as 2.28, with a sample size of 138. Patients were recruited to investigate the differential effects of peripheral vestibular function and cognition on spatial orientation (cross-sectional study design). General inclusion criteria were an age above 18 years and the ability to provide informed consent. Inclusion criteria for patients with chronic peripheral unilateral (Strupp et al., 2022) or bilateral (Strupp et al., 2017) vestibulopathy were applied according to the guidelines of the Bárány Society. Exclusion criteria were incomplete psychometric (MoCA, PHQ-9) or neuro-otological data, uncorrected hearing loss, relevant impairment of arm function due to, e.g., cerebellar ataxia, tremor, paresis, or orthopaedic disorders. Visual function was assessed during the neuro-orthoptic assessment using the Snellen-chart, and patients with insufficient visual function were excluded. Furthermore, patients with inadequate 3D-RWPT calibrations (which result in false-high angular deviations) were removed from the analysis. In total, 569 patients (302 females; mean age 62.77 ± 14.13 years, minimum age 19.8 years, maximum age 91.2 years) were included in the analysis, greatly exceeding the required minimum sample size.

The data protection clearance and Institutional Review Board of the Ludwig-Maximilians-University, Munich, Germany, approved the study (no. 094–10), and all patients gave informed consent. The study was performed in accordance with the ethical standards laid down in the 1964 Declaration of Helsinki and its later amendments. The development and validation of the 3D-RWPT was conducted in earlier studies (Gerb et al., 2023a; Gerb et al., 2022; Gerb et al., 2024a; Gerb et al., 2023b; Gerb et al., 2024b; Gerb et al., 2024c); the current analyses have not been described before.

### Neuro-otological testing

Clinical testing included a neurological and neuro-orthoptic examination, i.e., spontaneous and head-shaking nystagmus, ocular motor examination, fundus photography and adjustment of the subjective visual vertical (SVV), in order to detect central vestibular deficits and acute vestibular tonus imbalances (Brandt and Dieterich, 2017), bithermal water caloric testing, and standardized video-head-impulse-test measurements of the semicircular function in the high-frequency range using the EyeSeeCamHIT^®^ system (EyeSeeTec, Munich, Germany). Diagnoses of unilateral and bilateral vestibular hypofunction were made according to the international diagnostic criteria of the Bárány Society (Strupp et al., 2022; Strupp et al., 2017).

### Pointing task

**Figure 1:**
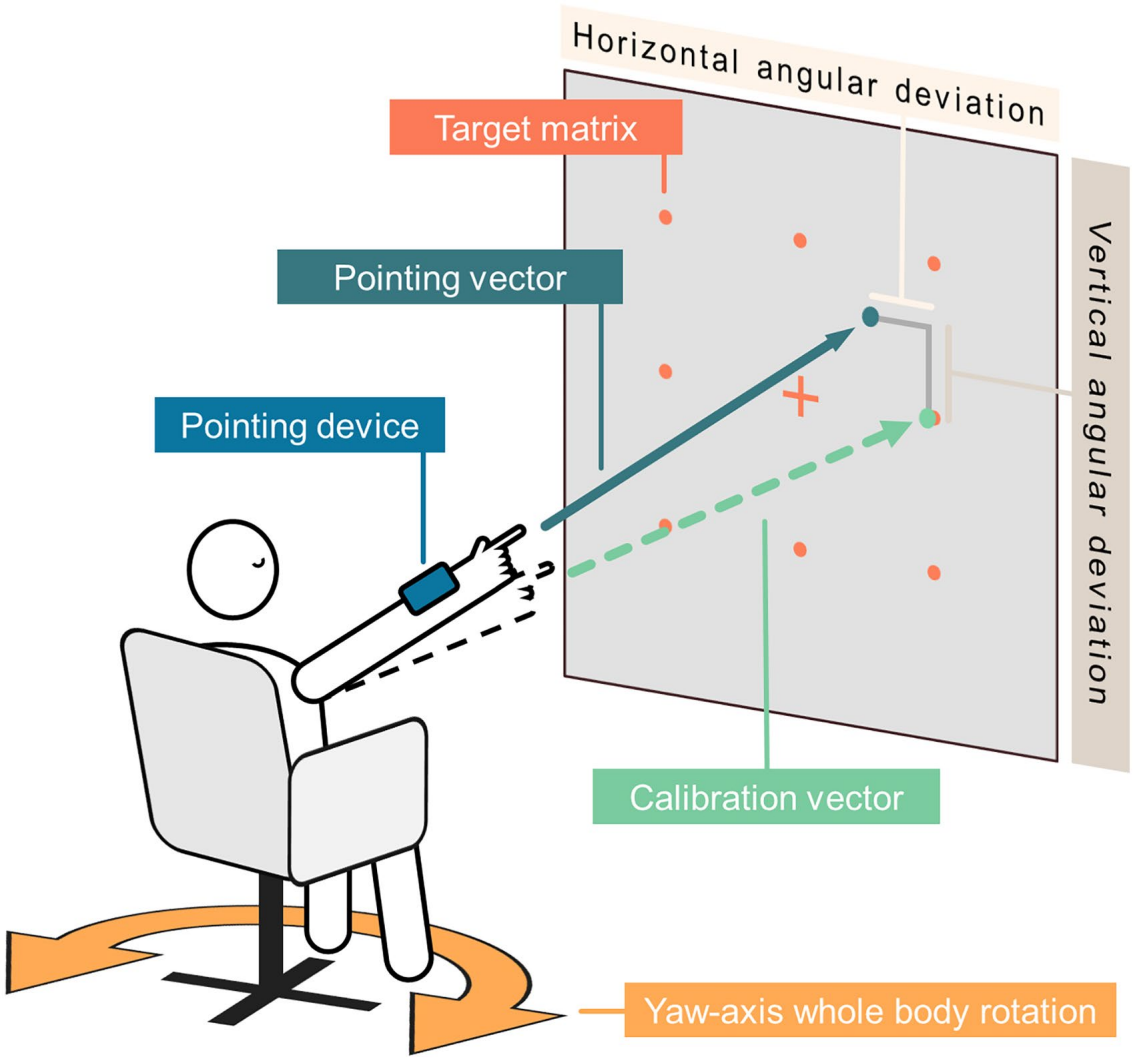
Schematic setup of the 3D-RWPT. The participant is seated roughly 2 m in front of a regular 9-dot target matrix marked with clearly visible dots (red), with a pointing device (blue) strapped to their forearm. Following two sets of calibrations with open eyes (green dotted arrow) the participant is then asked to point to each target in a newly randomized order with their eyes closed (reproduction task). Afterwards, the participant is passively rotated 90° to the non-hand-dominant side with their eyes open before performing the pointing task with their eyes closed (first transformation paradigm). The rotation back towards the starting position is performed without visual feedback, and the participant again points towards the targets (first post-rotation task). These two paradigms are then repeated towards the hand-dominant side (second transformation task, second post-rotation task). Based on the pointing vector (petrol arrow) recorded by the pointing device, angular deviation can be calculated in horizontal (=azimuth) and vertical (=polar) direction. All body rotations are around the participant’s yaw-axis (orange arrow) at 90°/s.

In the 3D-RWPT, the patients performed whole-arm pointing at a remembered array of nine targets from a seated position (Figure 1), first in a forward-facing body position (reproduction task), then after a passive body rotation 90° to the side (with visual feedback during the rotation, as a cognitively demanding mental transformation task), and then passively rotated back into the starting position (without visual feedback, i.e., relying on the correct integration of vestibular sensory information; post-rotation task) (Gerb et al., 2023a; Gerb et al., 2024a). All rotations were conducted at a rotational speed of 90°/s, resulting in a frequency of 0.25 Hz, which lies at the lower end of optimal vestibular stimulation (Haburcakova et al., 2012; Grabherr et al., 2008). Participants were aware that the rotation angle was 90°, both due to the visual feedback during the rotation as well as due to the oral test instructions given by the examiner. For each task, a computerized voice from a smartphone-based pointing device strapped to the hand-dominant forearm indicated the target in a newly-randomized order. The pointing vectors were recorded using the pointing device, and were compared to two sets of calibrations (egocentric/retinotopic and allocentric/world-based) to calculate the mean angular deviation in each plane for every paradigm. Larger deviations from the target position indicate lower pointing accuracy. Due to how the data is collected and processed, vectors were calculated in a spherical coordinate system, and the deviations were equally coded in the azimuth (i.e., horizontal) and polar (i.e., vertical) plane. Since this terminology stems solely from the mathematical concepts used in the data processing steps, we will use “horizontal” and “vertical” throughout the rest of the manuscript. Furthermore, we used the method described in Gerb et al. (2024a) to determine if participants employed a specific spatial encoding strategy. By subtracting the mean absolute task-specific deviation calculated with the world-based calibration from the mean absolute deviation calculated with the retinotopic calibration, a metric for the preferred spatial encoding strategy was derived. In short, this metric is negative for subjects performing the tasks mostly based on a retinotopically/egocentrically coded mental map, regardless of overall pointing accuracy (positive values: more world-based coding, negative values: more retinotopic coding, values around zero: no clear preference). So far, no definitive cut-off values for determining the employed spatial encoding strategy are available; in one previous study by our own group, patients with cognitive impairment (who are expected to use more egocentric/retinotopic spatial encoding) showed average values of −2° and lower in this metric, while values between −1° and +1° were interpreted as “no clear preference” (Gerb et al., 2024a).

### Statistical analysis

All data was irreversibly anonymized for data analysis and processed using Microsoft^®^ Excel (Version 2025) and JASP (Version 0.18.3, jasp-stats.org). For data description, mean values and standard deviation for continuous variables and absolute and relative frequencies for categorical variables were used. To assess the influence of each variable (MoCA, patient age, PHQ-9) and factor (vestibular function: normal, unilateral dysfunction, bilateral dysfunction; patient sex) on angular deviation, multiple mixed linear regression models (simultaneous predictor entry) were used for each task and plane. Residual correlation was assessed using the Durbin-Watson test, and parameter collinearity was ruled out using variance inflation testing.

## Results

Of the 569 patients, 67 (32 females, mean age 62.50 ± 14.22 years) fulfilled the diagnostic criteria for BVP, and 152 (74 females, mean age 63.13 ± 13.61 years) had chronic unilateral peripheral vestibular dysfunction (UVP; 78 left-sided, 74 right-sided), e.g., due to (fully compensated) unilateral vestibulopathy, Menière’s disease, or vestibular schwannoma. Vestibular testing results and psychometric scores from these cohorts can be seen in Table 1.

**Table 1:** Demographic, neuro-otological and psychometric testing results of the patient cohorts defined by vestibular function.

	BVP	UVP	No peripheral-vestibular deficit	Statistics (*p*-value adjusted for multiple testing)
N (of which females)	67 (32 females)	152 (74 females)	350 (196 females)	–
Age	62.50 ± 14.22 years	63.13 ± 13.61 years	62.67 ± 14.37 years	n.s.
Mean total caloric excitability	**2.27 ± 2.24 °/s**	**11.48 ± 5.72 °/s**	**20.13 ± 10.54 °/s**	**<0.001**
Mean vHIT gain at 60 ms (left side)	**0.33 ± 0.24**	**Left-sided UVP: 0.75 ± 0.30** Right-sided UVP: 0.88 ± 0.19	**0.95 ± 0.16**	**<0.001** (except for right-sided UVP vs. HC: n.s)
Mean vHIT gain at 60 ms (right side)	**0.35 ± 0.22**	Left-sided UVP: 0.82 ± 0.20 **Right-sided UVP: 0.70 ± 0.25**	**0.87 ± 0.16**	**<0.001** (except for left-sided UVP vs. HC: n.s)
MoCA-score	26.48 ± 3.06	26.43 ± 3.41	26.64 ± 3.09	n.s.
PHQ-9 score	6.60 ± 4.39	6.98 ± 4.39	6.38 ± 4.69	n.s.

Note that after subdividing the overall cohort by peripheral-vestibular function, all cohorts have comparable test scores in the cognitive screening test (MoCA) and in the dementia screening test (PHQ-9). Significant group differences are marked in bold print.

391 patients (211 females; mean age 58.79 ± 13.59 years) had normal test results in the MoCA dementia screening test, while 178 scored lower than 26 points (after correction for patient education level; 91 females, mean age 71.50 ± 11.06 years). Male and female patients did not diverge in their average MoCA-scores (Welch’s *t*-test: n.s.). The psychometric and neuro-otological testing results from these cohorts based on cognitive function can be seen in Table 2.

**Table 2:** Demographic, neuro-otological and psychometric testing results of the patient cohorts defined by cognitive function.

	Normal cognition	Cognitive impairment	Statistics (p-value adjusted for multiple testing)
N (of which females)	391 patients (211 females)	178 (91 females)	–
Age	**58.79 ± 13.59 years**	**71.50 ± 11.06 years**	**0.03**
Mean total caloric excitability	15.19 ± 10.33 °/s	16.47 ± 11.44 °/s	n.s.
Mean vHIT gain at 60 ms (left side)	0.83 ± 0.29	0.86 ± 0.26	n.s.
Mean vHIT gain at 60 ms (right side)	0.78 ± 0.25	0.78 ± 0.25	n.s.
MoCA-score	**28.25 ± 1.41**	**22.64 ± 2.91**	**<0.001**
PHQ-9 score	6.71 ± 4.96	6.93 ± 4.92	n.s.

Note that after subdividing the overall cohort by cognition, the cognitively impaired cohort has a higher average age, and substantially lower MoCA scores, while the vestibular function is comparable. Note that this binary classification of cognitive impairment was only used for this overview and the analysis of employed spatial encoding strategy, and more detailed MoCA-scores as a continuous metric were used in the regression models. Significant group differences are marked in bold print.

In all 3D-RWPT paradigms, the overall regression models were highly significant. In all models, the critical *F*-value based on the *a priori* power-calculation was reached. For all, the Durbin-Watson tests were around 2, as required. No parameter collinearity was detected (variance inflation testing: MoCA 1.30, age 1.29, PHQ-9 1.02, sex 1.03, vestibular function 1.01). The significant regression coefficients will be reported in the following paragraphs.

**Figure 2:**
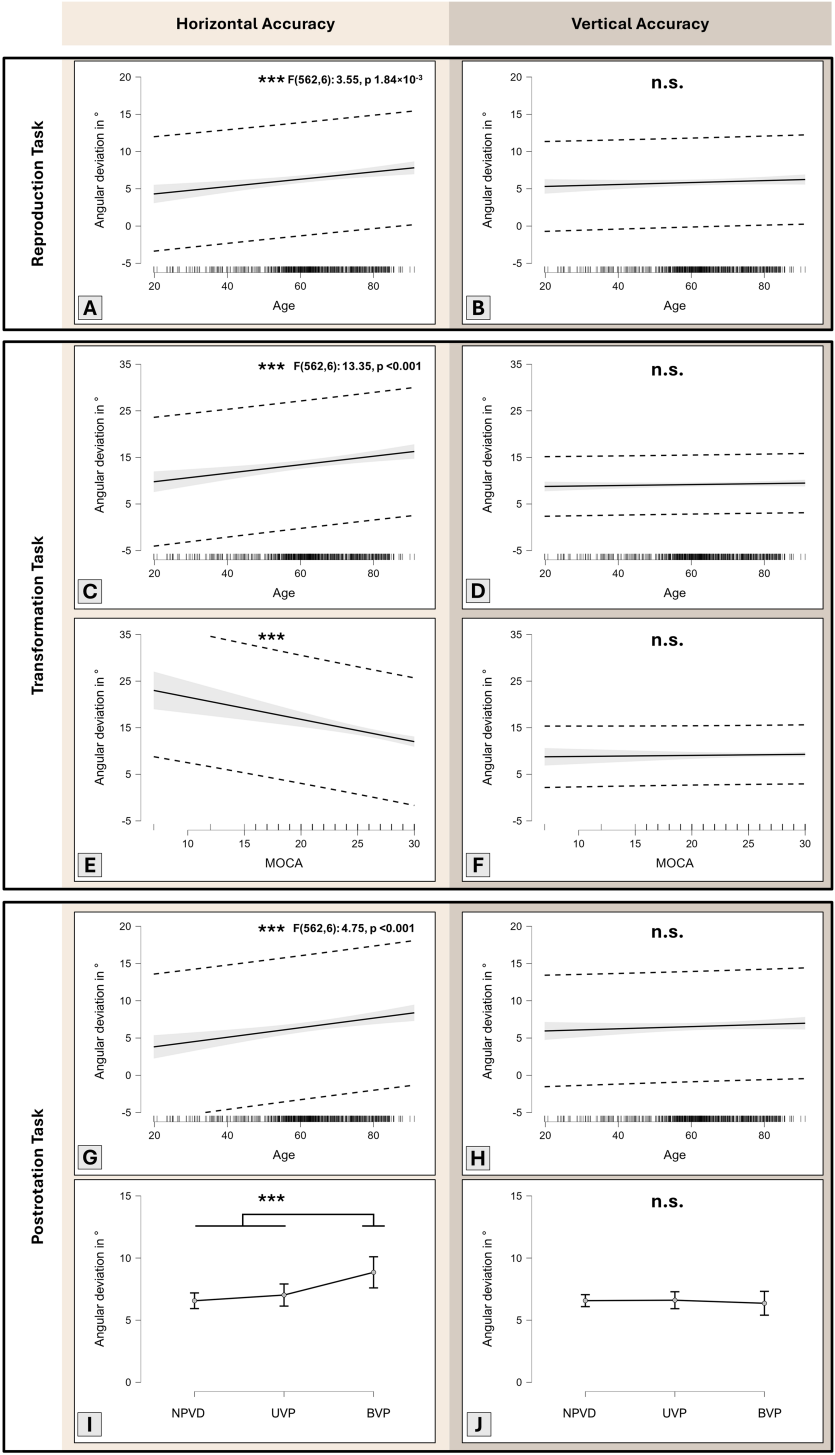
Marginal effects plots from 3D-RWPT performance (i.e., angular accuracy in ° (y-axis); lower values better) and covariates/factors. Grey area: 95% confidence interval; dotted lines: 95% prediction interval. In the initial reproduction task (which solely tests spatial memory), only patient age constituted a significant predictor of pointing accuracy in the horizontal **(A)**, but not the vertical plane **(B)**. In the (cognitively demanding) mental transformation paradigm, age again only constituted a significant predictor of pointing accuracy in the horizontal **(C)**, but not the vertical plane **(D)**; additionally, MoCA scores were a significant predictor of horizontal **(E)**, but not vertical **(F)** accuracy. In the postrotation task (which requires correct integration of vestibular sensory information), the same pattern of age effects prevails **(G,H)**. Furthermore, bilateral vestibular dysfunction (BVP) constituted a highly significant predictor for horizontal **(I)**, but not vertical **(J)** angular accuracy. No relevant parameter collinearity was detected using variance inflation testing (MoCA 1.30, age 1.29, PHQ-9 1.02, sex 1.03, vestibular function 1.01). Note that the 3D-RWPT uses a yaw-axis whole-body rotation (i.e., in the horizontal plane) as a stimulus, which might explain some of the findings of the transformation and the postrotation paradigms; in the initial reproduction task, however, no stimulus had been performed yet, and only spatial memory is being tested. MoCA: Montreal Cognitive Assessment, NPVD: no peripheral vestibular deficit, UVP: unilateral vestibulopathy, BVP: bilateral vestibulopathy.

In the reproduction task model summary horizontal plane: *F*(562,6) 3.55, p 1.84×10^−3^**; vertical plane: F(562,6) 2.70, p 9.41×10^−3^**), only age constituted a highly significant predictor for angular accuracy in the horizontal plane (t 3.78, coefficient 0.05 (95% CI: 0.02, 0.07, *p* < 0.001***, Figure 2A), but not in the vertical plane (n.s., Figure 2B). No other variables (PHQ-9, MOCA) or factors (patient sex, vestibular function) were significant predictors in the horizontal or vertical plane. Overall, the models were able to only partially explain pointing accuracy variability (adjusted R^2^ for horizontal accuracy: 0.19; vertical accuracy: 0.09).

In the (cognitively demanding) transformation paradigm (model summary horizontal plane: *F*(562,6) 13.35, *p* < 0.001***; vertical plane: F(562,6) 4.70, *p* < 0.001***), age and MoCA-score showed significant impact on the horizontal accuracy (age: t 3.88, coefficient 0.09 (95% CI: 0.05, 0.14), *p* < 0.001***, Figure 2C; MoCA: t − 4.72, coefficient −0.48 (95% CI: −0.68, −0.28), *p* < 0.001***, Figure 2E), while male patients generally outperformed female patients in both planes (horizontal: t − 2.48, coefficient −1.47 (95% CI: −2.63, −0.31), *p* 0.01*; vertical: t − 4.00, coefficient −1.10 (95% CI: −1.63, −0.56), *p* < 0.001***). No effect of age or MoCA was observable in the vertical plane (n.s., Figures 2D,F). The other variables (PHQ-9) and factors (vestibular function) did not constitute significant predictors in horizontal or vertical plane. Again, the models could only partially explain overall variability (adjusted R^2^ for horizontal accuracy: 0.35; vertical accuracy: 0.19).

In the postrotation task model summary horizontal plane: F(562,6) 4.75, *p* < 0.001***; vertical plane: F(562,6) 6.93, *p* < 0.001***), age and bilateral vestibular dysfunction (BVP) were highly significant predictors for horizontal accuracy (age: t 3.87, coefficient 0.06 (95% CI: 0.03, 0.10), *p* < 0.001***, Figure 2G; BVP: t 3.49, coefficient 2.29 (95% CI: 1.00, 3.57), *p* < 0.001***, Figure 2I, but not for vertical accuracy (n.s., Figures 2H,J). Male patients outperformed female patients in the vertical plane (t − 6.07, coefficient −1.94 (95% CI: −2.57, −1.32), *p* < 0.001***). All other variables or factors were not significant predictors. Similar to the other paradigms, the models were only able to partially explain overall variability (adjusted R^2^ for horizontal accuracy: 0.20; vertical accuracy: 0.17).

### Subgroup analysis on ageing effects

**Figure 3:**
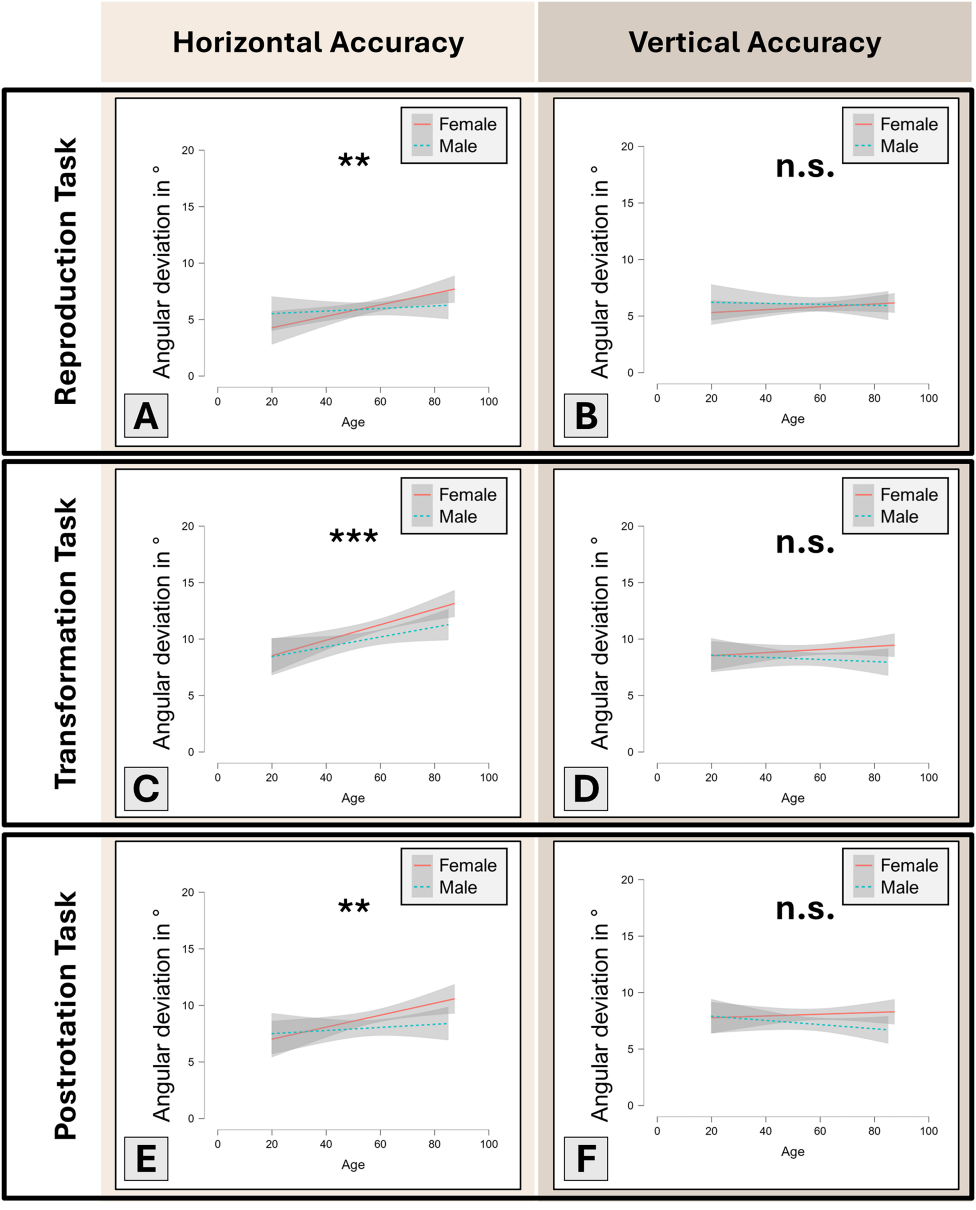
Linear correlation plots between age and 3D-RWPT performance (i.e., angular accuracy in ° (y-axis); lower values better) in participants with normal peripheral-vestibular testing results and normal cognition (*n* = 278). Significant linear correlations are marked with asterisks (*p* < 0.01 **, *p* < 0.001 ***). Grey area: 95% confidence interval, red line: female participants, teal dotted line: male participants. In all horizontal paradigms **(A,C,E)**, higher age was correlated with worse performance (i.e., larger angular deviation). In all vertical paradigms **(B,D,F)**, age did not correlate with 3D-RWPT performance.

In an additional analysis, we excluded all patients with persisting peripheral vestibular dysfunction (UVP, BVP) as well as patients with suspected cognitive impairment in the dementia screening test (MoCA-score < 26 pts. after correction for patient education level), and investigated the effects of age on each 3D-RWPT paradigm. In total, 278 patients (167 females; mean age 57.61 ± 15.20 years) fulfilled these requirements. In the reproduction task (i.e., the spatial memory paradigm), age showed a positive correlation with horizontal angular deviation (Spearman’s rho 0.19, p 1.72 × 10^−3^**, Fisher’s z 0.19, Figure 3A), but not with vertical angular deviation (n.s., Figure 3B).

In the transformation task, age showed a positive correlation with horizontal angular deviation (Spearman’s rho 0.24, *p* < 0.001***, Fisher’s z 0.25, Figure 3C), but not with vertical angular deviation (n.s., Figure 3D). Similarly, in the postrotation task, age showed a positive correlation with horizontal angular deviation (Spearman’s rho 0.17, p 3.43 × 10^−3^**, Fisher’s z 0.18, Figure 3E), but not with vertical angular deviation (n.s., Figure 3F).

No sex differences were found in the initial reproduction task (Welch’s *t*-test: n.s.). In both the transformation (Welch’s *t*-test: t 1.72, p 0.04*, Cohen’s d 0.21) and the post-rotation task (Welch’s *t*-test: t 1.81, p 0.04*, Cohen’s d 0.22), male participants outperformed female participants in the horizontal plane, while no sex differences were observable in the vertical plane (Welch’s *t*-test: n.s.).

**Figure 4:**
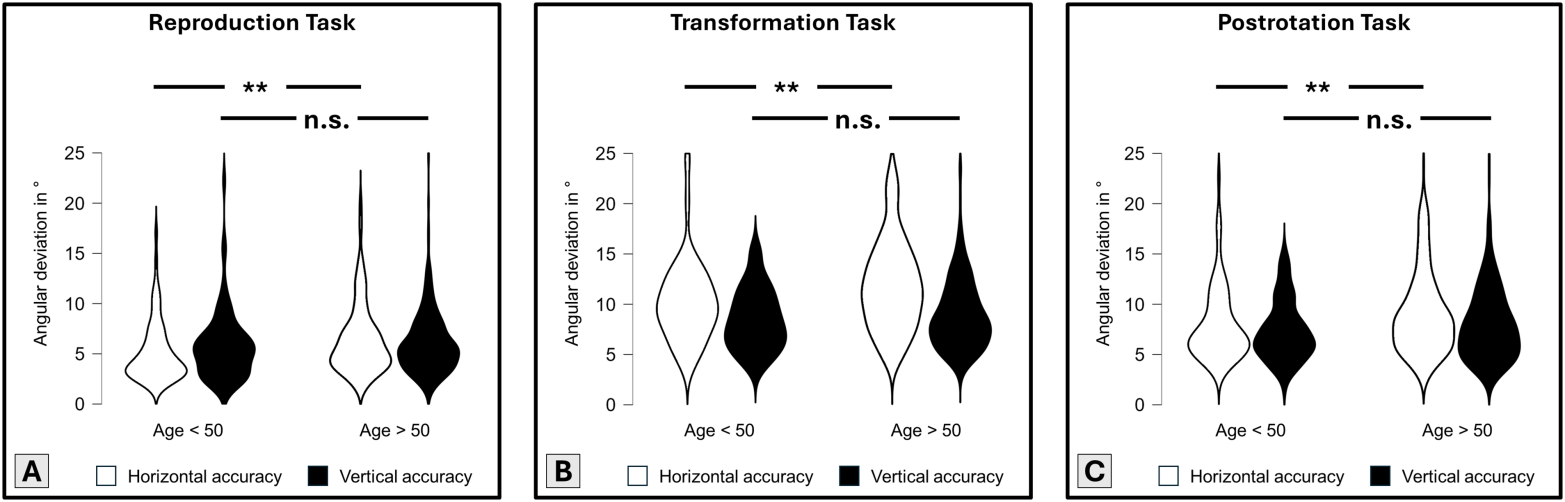
Cohort analysis for young (below 50 years, *n* = 71) and old (above 50 years, *n* = 207) participants with normal peripheral-vestibular testing results and normal cognition, and 3D-RWPT performance [i.e., angular accuracy in ° (y-axis)]; lower values better; **(A)** reproduction paradigm; **(B)** transformation paradigm; **(C)** post-rotation paradigm. Significant group differences (independent samples *t*-test) are marked with asterisks (*p* < 0.01 **). In all horizontal analyses (white violin plots), the younger cohort outperformed the older cohort; in all vertical analyses (black violin plots), no group differences were observable.

Additionally, we divided this patient cohort into young patients (age < 50 years; *n* = 71, 39 females, mean age 36.27 ± 8.24 years) and old patients (age > 50 years; *n* = 207, 128 females, mean age 64.93 ± 8.77 years). In all horizontal paradigms, young patients outperformed old patients (reproduction: Welch’s *t*-test: t − 2.91, p 2.12 × 10^−3^**, Cohen’s d − 0.38; transformation: t − 2.55, p 6.03 × 10^−3^**, d − 0.35; postrotation: t − 2.76, p 3.23 × 10^−3^**, d − 0.35). Again, the groups did not diverge in their vertical accuracy (Welch’s *t*-test n.s.; Figure 4).

### Spatial encoding in cognitive impairment

Age-corrected ANCOVA revealed a shift towards egocentric/retinotopic spatial encoding in patients with cognitive impairment in all 3D-RWPT paradigms (reproduction, horizontal plane: *F*(562,6) 5.06, p_Tukey_ 0.02*, vertical plane F(562,6) 3.95, p_Tukey_ 0.05*; transformation, horizontal plane: F(562,6) 5.61, p_Tukey_ 0.02*, vertical plane F(562,6) 16.28, p_Tukey_ < 0.001***; postrotation, horizontal plane: F(562,6) 4.09, p_Tukey_ 0.04*, vertical plane F(562,6) 7.40, p_Tukey_ 6.70 × 10 ^−3^**). No clear spatial encoding strategy patterns were observable in the vestibular cohorts.

## Discussion

In a large patient cohort from a tertiary interdisciplinary vertigo and balance center, higher age significantly reduced horizontal pointing accuracy in a test of sensorimotor spatial abilities, while vertical angular accuracy remained unaffected. This was observable in all paradigms of the 3D-RWPT, including the initial reproduction paradigm which solely required pointing at remembered targets in a stationary sitting participant. Furthermore, previous findings from the 3D-RWPT could be confirmed, namely, the differential effects of vestibular and cognitive impairment on spatial abilities (Gerb et al., 2024a; Gerb et al., 2024c). This is also in line with earlier findings on the morphological configuration of 3D-RWPT performance as a holistic measure of spatial representation, which revealed horizontal (but not vertical) deficiencies in patients with cognitive decline (see Figure 4 in Gerb et al., 2023b). The observed effects seem to indicate that horizontal and vertical spatial perception show distinct associations with patient age: horizontal spatial accuracy decreases with higher age, while vertical accuracy does not. While ageing-related cognitive or vestibular impairment might constitute a potential confounder in the transformation and post-rotation paradigms of the 3D-RWPT, the initial reproduction paradigm involves neither a cognitively demanding mental transformation nor the need for correct integration of vestibular input. Furthermore, two additional confirmatory analyses were conducted in participants without cognitive or vestibular impairment (Figures 3, 4) which showed the same pattern.

Aging, cognitive decline, and peripheral vestibular loss all impaired horizontal but not vertical orientation. Even in a purely static spatial memory task, horizontal accuracy decreased, while vertical accuracy did not. It is noteworthy that the horizontal body rotation used in the 3D-RWPT seemed to induce plane-specific spatial disorientation, rather than globally decreased pointing accuracy. To some extent, this constitutes a limitation of the 3D-RWPT, which does not include a pitch plane or roll plane stimulus. Here, future iterations of the test could include three-dimensional changes of body position using a Space Curl system (Bergmann et al., 2015).

While a significant effect of chronic BVP on 3D-RWPT performance in the post-rotation task was demonstrated for the horizontal plane, confirming our previous investigation (Gerb et al., 2024a), no clear effect was seen in the UVP cohort. For BVP, substantial effects on human and rodent navigation have been demonstrated in previous studies (Yoder and Taube, 2014; Zwergal et al., 2024; Brandt et al., 2005; Kremmyda et al., 2016; Dobbels et al., 2019). While UVP has shown distinct spatial memory deficits in animal models (El Mahmoudi et al., 2023) and patients (Hüfner et al., 2007), the deficits are typically less pronounced in UVP than in BVP (Zwergal et al., 2024; Ahmad et al., 2022). Not all studies could reproduce spatial impairment in UVP patients, especially in cases with milder loss of peripheral vestibular function (Grabherr et al., 2011) [for a recent review, see (Oh et al., 2024)]. Furthermore, the side of UVP seems to play a role for spatial orientation (Nguyen et al., 2021). The lack of clear findings in our UVP cohort could be due to the analysis approach, which pooled left- and right-sided UVP and left- and right-sided whole-body rotation (i.e., no side-specific analysis). This might explain why findings from Borel et al. (2014) could not be reproduced in the current study. Here, further studies which solely focus on a larger number of UVP patients and employ a more detailed data analysis centered around this specific research question are needed.

Previous studies have shown how humans are generally better at horizontal rather than vertical spatial tasks (Zwergal et al., 2016; Du and Mou, 2022; Hinterecker et al., 2018; Brandt et al., 2015; Ertl et al., 2019), leading to the concept of a human anisotropy of spatial encoding. Some of these studies, however, were conducted in rather young participants, e.g., university students (Du and Mou, 2022). To our knowledge, the current investigation is the first large-scale, clinical cohort study which assessed age-related changes of vertical and horizontal spatial accuracy. The only other pointing study investigating age effects described no accuracy differences between 12 young and 12 older adults (Lemay et al., 2004). However, the latter results (Lemay et al., 2004) are difficult to compare to our findings. Not only did the participants have visual feedback in all but one of their experimental paradigms, the authors also mainly focused on movement kinematics, and only reported radial accuracy in detail. While separate analyses of vertical and horizontal accuracy were conducted, they were unfortunately only briefly mentioned in a footnote. Unlike the results of Lemay et al. (2004), we could show that even after exclusion of participants with cognitive impairment or vestibular dysfunction, young participants outperformed older participants in the horizontal, but not the vertical plane. Importantly, this was again already observable in the initial reproduction task, which does not require spatial updating, but solely tests two-dimensional horizontal and vertical spatial memory. Here, daily training effects might play a role: younger people tend to be more active than older people, including more daily (horizontal) locomotion. Horizontal physical exercise such as fast-paced walking has been linked to hippocampus volume increase, and improved spatial memory (Erickson et al., 2011). Interestingly, long-term physical exercise in the vertical plane, e.g., in professional rock climbers, has been shown to induce structural changes in the cerebellum (Di Paola et al., 2013). Potentially, a predominantly horizontal training bias through every-day locomotion might explain our findings. Interestingly, experiments designed to modulate spatial perception have been shown to induce long-lasting distortions of egocentric reference frames (Dupierrix et al., 2009). Vice versa, one can postulate that everyday (predominantly horizontal) locomotion and spatial interaction with the real three-dimensional world similarly adjusts and calibrates spatial reference frames.

This increasing anisotropy of age-dependent impairment of horizontal versus vertical spatial orientation might be in line with two different modes of spatial encoding, as proposed in Brandt and Dieterich (2016). Here, the authors introduced the concept of how vestibular input from the otoliths in stationary subjects contributes to two-dimensional egocentric spatial orientation, while three-dimensional allocentric spatial orientation (in mobile subjects) utilizes input from otoliths and semicircular canals. While this fits previous research, there is no clear understanding of the structural and functional integration of vestibular input into vertical spatial orientation (for a review, see Jeffery et al., 2013), for the role of otolith sensory information for spatial navigation (see also, Yoder and Taube, 2014; Xie et al., 2017). Importantly, in all experimental paradigms of the 3D-RWPT, pointing in the vertical plane can arguably be achieved by an egocentric/static relationship between the body and the target array, while pointing in the horizontal plane quintessentially requires mental updating of the relationship between the body and the environment due to the repeated horizontal whole-body rotations. This can only be achieved by a dynamic strategy which incorporates allocentric/world-based cues (Brandt and Dieterich, 2016). Importantly, the terminology used for the spatial operational modes introduced by Brandt and Dieterich is slightly different from the terminology we previously used to describe the 3D-RWPT: an egocentric pointing strategy in the 3D-RWPT is based on retinotopic visuospatial encoding (i.e., in subject-centred coordinates, hence referred to as “egocentric”). Conceptually, a strict dichotomous separation of operational modes is unlikely, since both modes of operation and reference frames would probably be integrated task-specifically. In the current dataset, it seems like the static spatial operational mode remains stable independent of patient age, while the dynamic spatial operational mode required for updating the mental model of body-in-space appeared to be more error-prone in higher age. This is in line with prior research on human navigation: ageing impairs allocentric navigation (Moffat and Resnick, 2002; Moffat et al., 2006; Harris et al., 2012; Colombo et al., 2017). In our dataset, cognitive impairment additionally disturbed allocentric spatial encoding even after correcting for participant’s age, resulting in a predominantly egocentric spatial encoding strategy in patients with pathological dementia screening tests. Again, this fits previous studies which could show that allocentric spatial encoding is commonly impaired in Alzheimer’s disease (Ruggiero et al., 2020; Serino et al., 2015), and confirms previous findings by our own group in patients with cognitive decline (Gerb et al., 2024a; Gerb et al., 2024c). It should be noted that this shift towards egocentric spatial encoding was observable in all 3D-RWPT subtasks and in both horizontal and vertical planes, indicating a global rather than a plane-specific phenomenon.

The scores from a depression screening test (PHQ-9) did not constitute significant predictors of angular accuracy in the 3D-RWPT in any of the regression models. While some previous studies have found spatial memory impairment in depressive patients (Erle et al., 2019; Asthana et al., 1998), a meta-analysis by McDermott and Ebmeier (2009) found no evidence for a correlation between depression severity and visuo-spatial memory. However, it should be noted that in our cohort no further psychiatric assessments beyond the self-reported screening test used were performed.

The conclusion that spatial memory in the vertical plane remains unaffected by ageing, cognitive decline, and vestibular impairment is based on a single three-dimensional pointing test. Although the data is compatible with that of earlier studies in which spatial abilities were mainly tested in the horizontal plane (for a recent meta-analysis, see Plácido et al., 2022), further studies are required to generalize the reliability and robustness of vertical orientation in neurological conditions which are known to induce predominantly pitch-plane symptoms, e.g., vertical nystagmus syndromes (Dieterich et al., 1998). Furthermore, it should be noted that spatial abilities exhibit a generally large interindividual variance (Hegarty et al., 2006; Weisberg et al., 2014). This is partially reflected in the performance metrics of the linear regression models, which typically indicated a sub-par explanation of individual variance of pointing accuracy. Importantly, this is not unexpected in the field of spatial-behavioral research. Considering the context of lifelong spatial learning, these individual differences might play a role. However, we tried to mitigate this confounder by choosing a large-scale approach in the current study, resulting in a cohort orders of magnitude larger than previous investigations of vertical spatial abilities in humans (Zwergal et al., 2016; Du and Mou, 2022; Lemay et al., 2004). Still, future research, using both animal models of vertical navigation as well as neuroimaging studies in humans, is needed to explain why humans exhibit not only an anisotropy of spatial memory, but also how interindividual differences affect this anisotropy, and why vertical spatial abilities seem to remain mostly unaffected by vestibular dysfunction, cognitive impairment, or ageing.

## Data Availability

The raw data supporting the conclusions of this article will be made available by the authors, without undue reservation.
